# The scalp as a donor site for skin grafting in burns: retrospective study on complications

**DOI:** 10.1186/s41038-016-0042-z

**Published:** 2016-07-13

**Authors:** Dorota Teresa Roodbergen, Adrianus Fredericus Petrus Maria Vloemans, Zjir Mezjda Rashaan, Jacob Cornelis Broertjes, Roelf Simon Breederveld

**Affiliations:** 1 Burn Centre, Red Cross Hospital, Vondellaan 13, 1942 LE Beverwijk, The Netherlands; 2 Department of Surgery, Red Cross Hospital, Beverwijk, The Netherlands; 3 Department of Surgery, University Medical Centre Leiden, Leiden, The Netherlands

**Keywords:** Burn, Scalp, Donor site, Graft

## Abstract

**Background:**

Split skin grafting (SSG) is the cornerstone in the treatment of deep burns and large skin defects. Frequently used donor sites are the thigh, abdomen and buttocks. The scalp is less common while considered as a reliable donor site. Advantages are a large surface area, rapid wound healing, cosmetically favourable results and multiple harvests from the same donor site. Complications include scab formation, chronic folliculitis and alopecia but have been recorded sporadically in previous studies. This article evaluates the complication rate of the scalp donor site in the treatment of deep burns in the Beverwijk Burn Centre.

**Methods:**

A retrospective study was performed of all patients who received a skin graft from the scalp at the Beverwijk Burn Centre between January 2004 and December 2012. Data were collected from medical files of included patients, including gender, age, type of burn (scald, flame, other) and total body surface area (TBSA) burned at the time of first surgery. Postoperative variables were healing time of the donor site and incidence of complications. During follow-up, the incidence of late complications was reviewed.

**Results:**

A total number of 105 grafts were analysed in 93 patients: 58 males (62 %) and 35 females (38 %), with a median age of 2 years and 3 months old. Of the patients, 30 (32 %) had flame burns and 57 (61 %) had scald burns. Eighty-seven percent of patients had a TBSA burned of 5 % or less. All donor sites healed within 14 days. No alopecia or scar hypertrophy developed at the donor sites. Two patients (2.2 %) developed folliculitis; one patient (1.1 %) showed scab formation.

**Conclusions:**

The scalp as a donor site in our Burn Centre shows a comparable short-term complication rate to the previous literature, with quick healing and no long-term complications. Therefore, we propose the consideration of the scalp as a primary donor site, especially in young children, where the scalp offers a larger donor site area than the buttocks or thighs.

## Background

Split-thickness skin grafting (SSG) is the cornerstone in the treatment of deep partial-thickness burns with a large risk of scar formation, full-thickness burns and large skin defects. The common donor sites are the thigh, abdomen and buttocks. In the treatment of deep partial-thickness burns, skin grafting is usually carried out 10 to 14 days post-burn in the Beverwijk Burn Centre.

The scalp as a donor site is less common, while it is considered a reliable donor site since its first use in 1964 [1]. The advantages are less painful compared to other donor sites [2–4], cosmetically favourable results as the re-growth of hair conceals the donor site [3–8] and a relatively large surface area when applied in children [5, 8]. Other reported advantages are the faster epithelialisation of the scalp [9] and good colour matching [10] with the skin of the face. The faster epithelialisation is due to the high amount of dermal appendages—and thus, a substantial supply of follicular epithelial stem cells—and the scalp’s rich vascularity [3, 4, 6, 7, 9]. This allows for multiple harvests from the same site [3, 5–7, 9, 11, 12]. The good colour matching with the face makes the scalp an ideal donor site when treating facial burns [3–7, 10]. Possible complications include folliculitis, scab formation and alopecia [3–7, 11, 12]. These complications have been recorded with a rate of 1.5 to 7.3 % in previous studies [3–7, 11, 12].

Chang et al. [12] define the ideal donor site as “easy harvest, minimal bleeding, easy postoperative care, availability of repeat harvest, reasonable percentage of wound coverage, rapid wound healing, minimal interference with rehabilitation and minimal adverse effects.”

This article evaluates the complications of the scalp as a primary donor site in the treatment of burns in the Beverwijk Burn Centre.

## Methods

### Patients

This retrospective study was conducted in the Red Cross Hospital in Beverwijk, The Netherlands. All patients who received a split-thickness skin graft from the scalp as a donor site between January 2004 and December 2012 were included. All patients were treated per in-hospital protocol, as described below.

### The in-hospital protocols

#### Determination of donor site

If the wound is judged to be indicated for surgery, usually around the 8^th^ to 12^th^ day post burn, the surgery and donor site are discussed with the patient or parents. Especially in children, the scalp is the first choice of donor site. When patients or parents strictly object after explanation and refuse to give their informed consent, a different donor site is chosen.

In adults, the scalp is the primary choice of donor site in burns above the clavicles because of the good colour match.

Contraindications for the use of the scalp are conditions in which impaired wound healing of the scalp is expected, for instance, atrophy of the skin of the scalp, burns of the scalp or pre-existent skin conditions of the scalp such as folliculitis, dermal mycosis or eczema.

#### Skin grafting

**Fig. 1:**
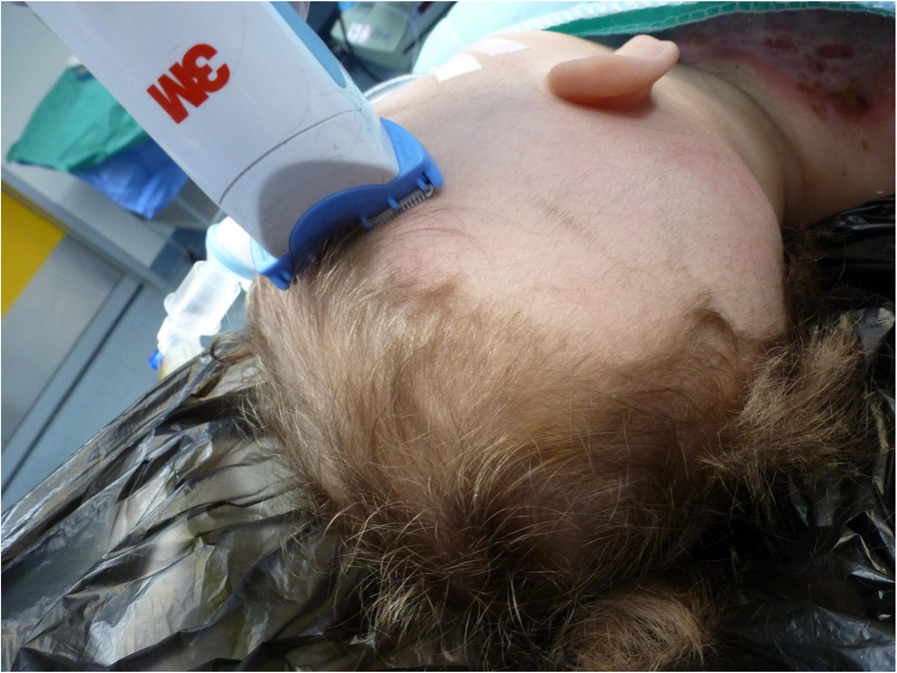
Preparation of the donor site. Shaving of the scalp

**Fig. 2:**
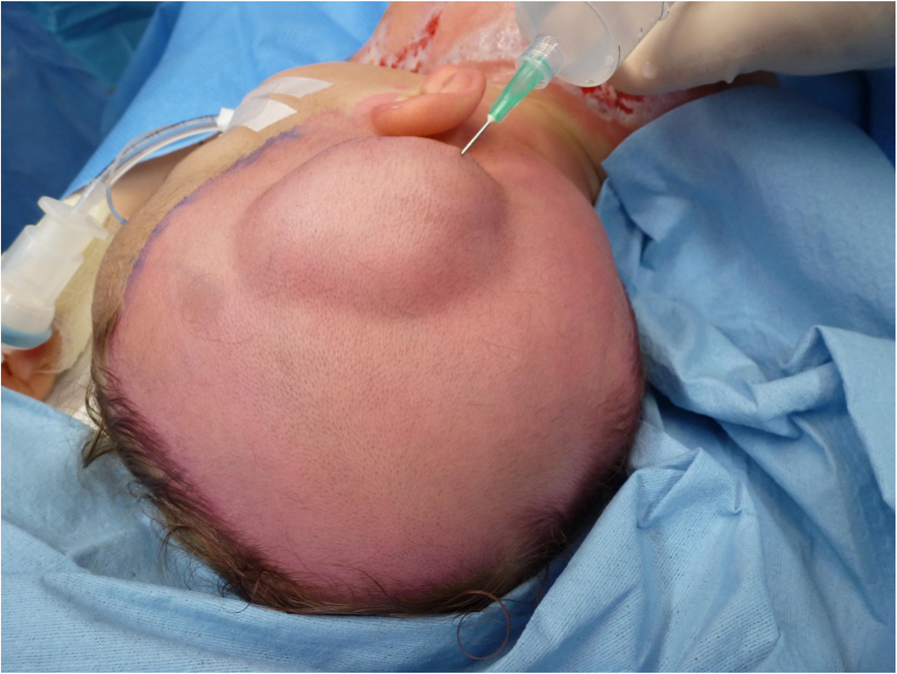
Preparation of the donor site—continued. Subgaleal infiltration of the donor site, with indication of hairline visible

**Fig. 3:**
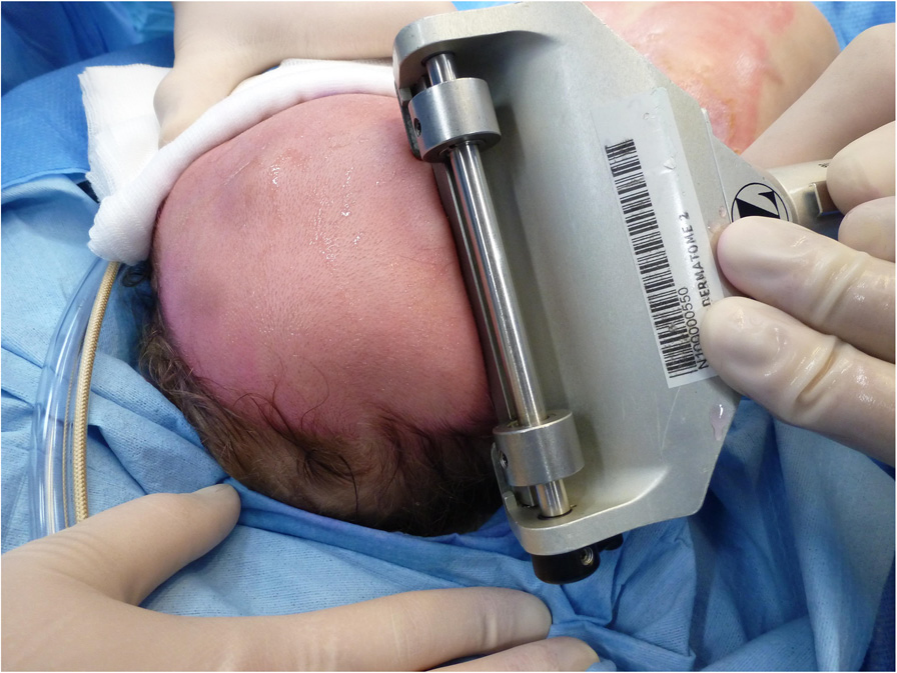
Harvesting procedure. Harvesting of the split-thickness skin graft

**Fig. 4:**
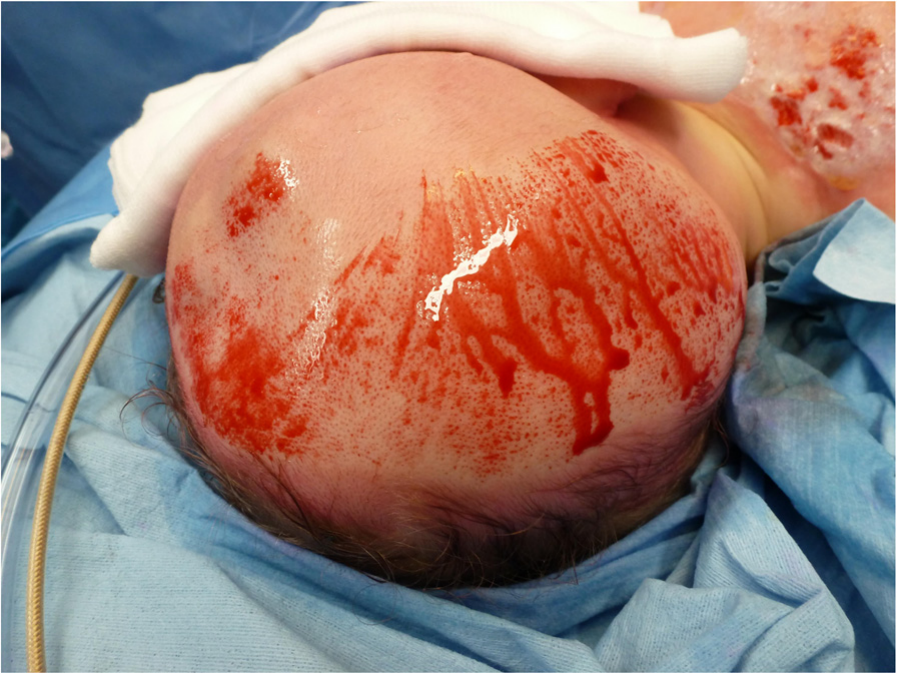
Harvesting procedure—continued. Aspect of the donor site after superficial harvesting, with intact hair follicles

**Fig. 5:**
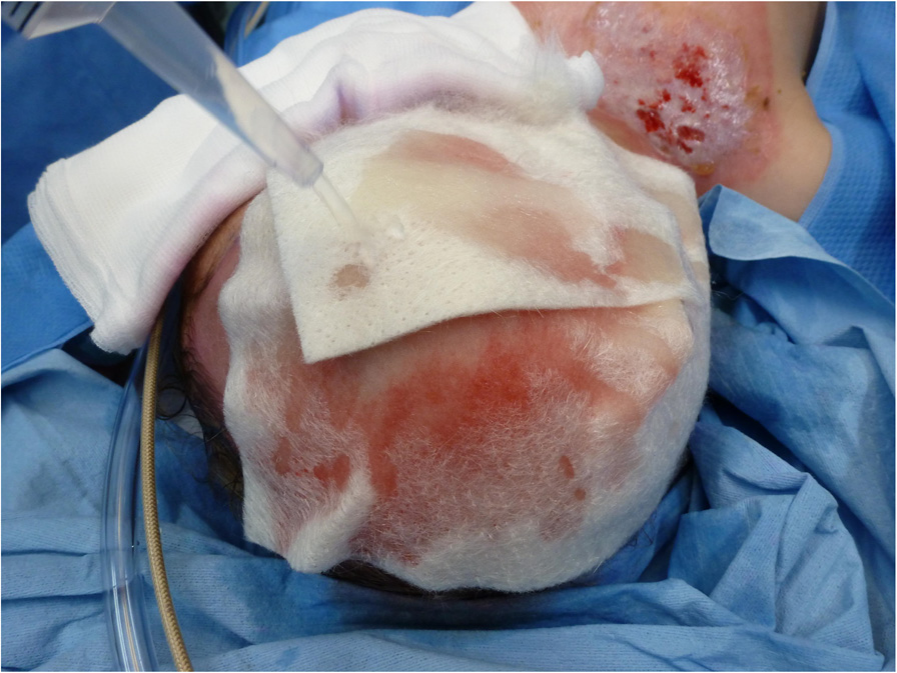
Haemostasis. Application of adrenalin solution on the alginate dressing for haemostasis

**Fig. 6:**
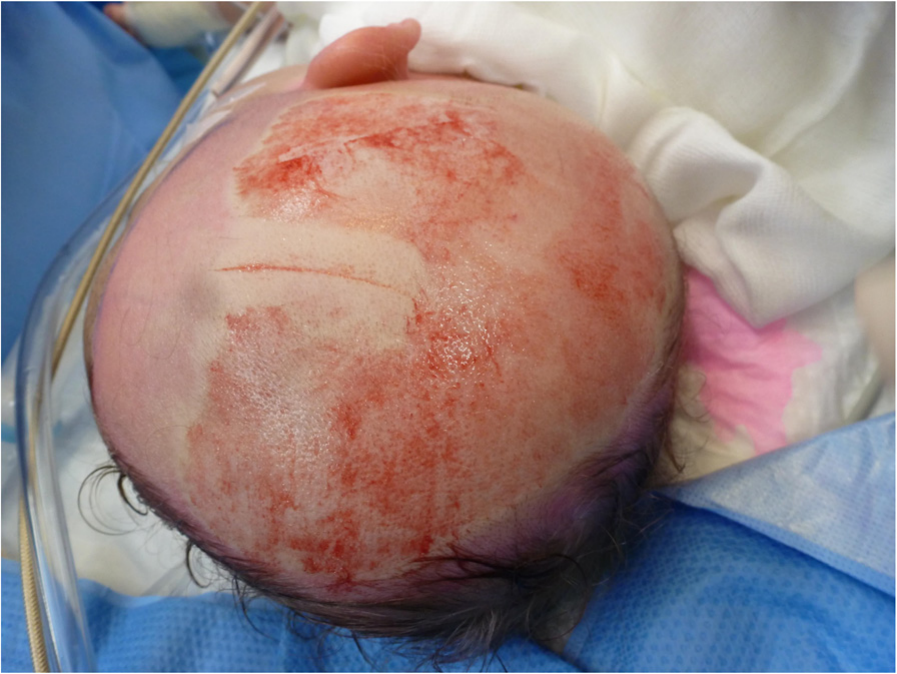
Aspect of donor site after hemostatic dressing. Note the pale aspect of the surrounding intact skin due to the applied adrenaline solution

The procedure of skin grafting from the scalp is performed by an experienced burn surgeon. First, the scalp is shaved, and if there is any risk of harvesting of the skin outside the boundary of the scalp, the hairline is indicated with a surgical marker (Fig. 1). Thereafter, the donor site is disinfected with a chlorhexidine solution (0.5 % chlorhexidine with 70 % alcohol) and infiltrated with a sterile physiological saline solution (NaCl 0.9 %) in the subgaleal space to obtain a cushion that allows harvesting of a wide strip of skin (Fig. 2). The thin autograft is then harvested (Figs. 3 and 4) with a Zimmer^®^Air Dermatome (Zimmer Inc. Warsaw, IN, USA). Haemostasis is achieved by a temporary alginate dressing soaked in adrenaline solution: 10 mg adrenaline in 1 L NaCl 0.9 % (Figs. 5 and 6).

#### Dressing of the donor site

**Fig. 7:**
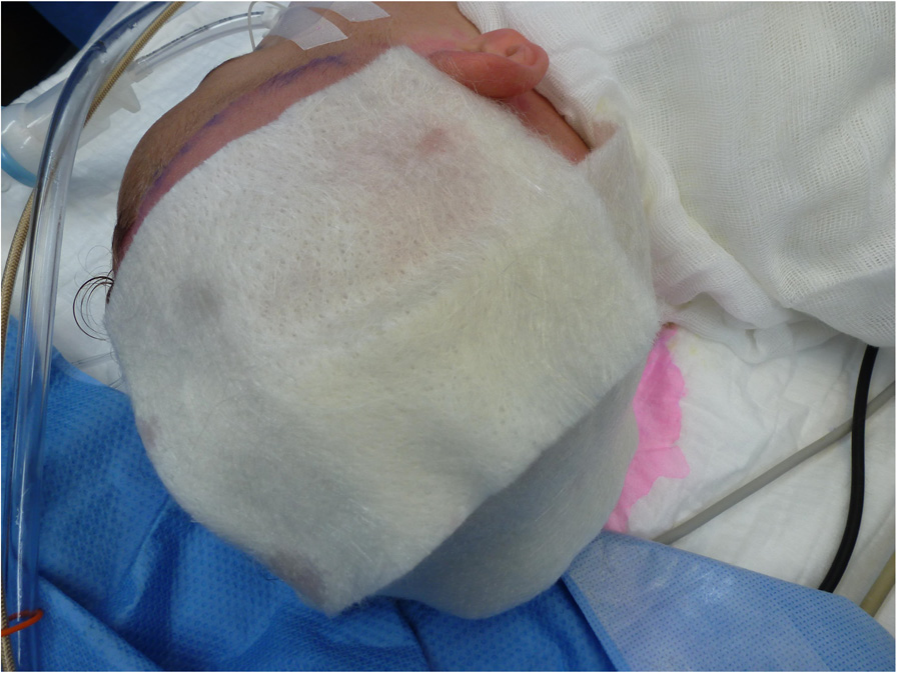
Definitive alginate dressing. This dressing will adhere to the donor site and form a crust, allowing the donor site to heal underneath with minimal pain

After haemostasis, a definitive alginate dressing is applied to the donor site (Fig. 7). This is covered with absorbent cotton gauze and secured with elastic bandage and an elastic stocking.

In the first 3 to 5 days postoperatively, the exudate from the donor site keeps the alginate moist, enabling the alginate dressing to migrate or dislodge. During this period, the outer bandages are necessary to protect the donor site and secure the alginate dressing. If the outer bandage and gauzes are moist, only these are changed, leaving the alginate dressing in place. After this period, the alginate dressing forms a dry adherent crust and the upper bandages and absorbent gauze can be removed.

The alginate crust detaches spontaneously from the donor site when healed.

#### Dermatome setting

**Fig. 8:**
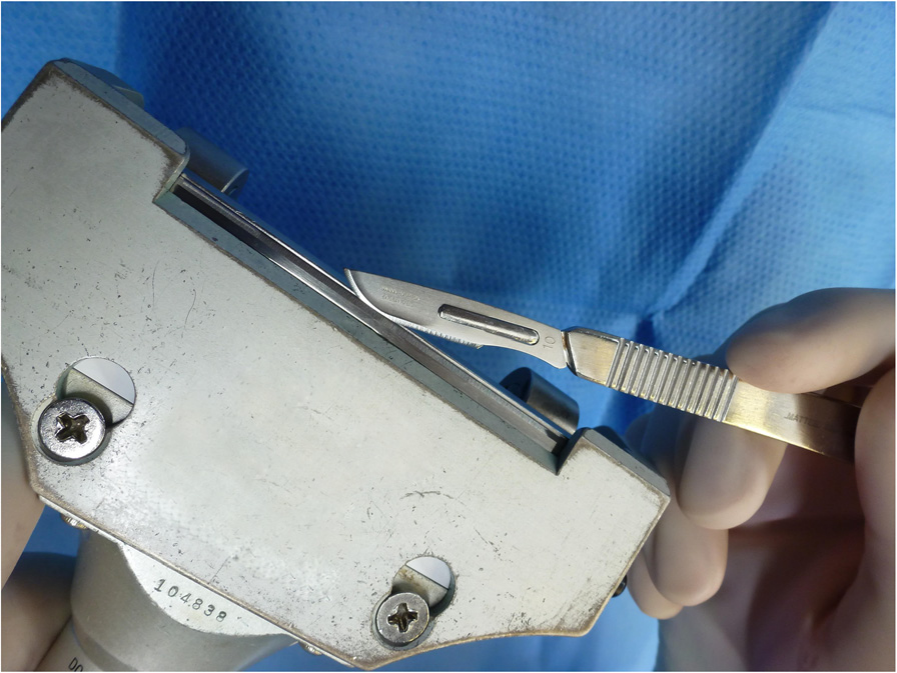
Checking the dermatome setting with a scalpel blade. The edge of the blade should just fit in between the blade of the dermatome and the guard. If the rest of the scalpel blade fits in between, the harvesting will be too deep

In literature, the thickness of the harvested skin graft is usually indicated by the setting of the dermatome, not by actual measurement of the graft. In practice, we have found that the thickness of the graft may vary with the same dermatome setting. We, therefore, insert a scalpel blade between dermatome blade and guard before harvesting, with the edge of the blade just fitting in between, to check the setting of the dermatome and to adjust if necessary (Fig. 8) [2]. In practice, we found that by adjusting the dermatome setting to the thickness of the scalpel blade, the setting on the dermatome varies between around 0.008 and 0.012 in. or 0.20–0.30 mm.

#### Treatment of complications

The primary treatment of the occurring folliculitis or scab formation is conservative. The affected area, including a wide margin of 2 cm, is shaved daily. The scalp is then rinsed with a chlorhexidine solution and a topical antiseptic applied. This process is repeated daily until the folliculitis or scab formation recedes.

#### Appraisal of healing of the donor site

The definition of donor site healing is an epithelialisation of 95 % of the donor site. This is appraised by a medical professional, i.e. burn physician, on a routine outpatient check-up at around 2 weeks postoperatively, or in the clinic at detachment of the alginate crust.

### Data

Baseline characteristics were collected from medical charts, surgery reports, discharge letters and photos taken during admission and follow-up, including gender, age, type of burn (scald, flame, other) and total body surface area (TBSA) involved at the time of the first surgery. Split-thickness skin grafting was indicated in deep partial- to full-thickness burns. According to our protocol, these are defined as burn wounds unlikely to have healed within 21 days post-burn, assessed on the 10^th^ to 14^th^ day post-burn.

Postoperative variables were the time of healing of the donor site and the incidence of donor site complications, like folliculitis and scab formation. During the follow-up period in the outpatient clinic, the incidence of late donor site complications like alopecia and the development of scar hypertrophy was registered.

## Results

A total number of 105 grafts in 93 patients was analysed (Table 1). Over half of these were male patients (63 %). The mean age was 2 years and 3 months, and 66 % of all patients were 5 years of age or younger. The majority of burns was represented as scald burns (61 %). Most patients (87 %) had a small TBSA involved of 5 % or less. TBSA: total body surface area.

**Table 1:** Patient characteristics

Total	*n* = 93
Gender	
Male, *n*(%)	58 (62.4 %)
Female, *n*(%)	35 (37.6 %)
Cause of burn	
Scald, *n*(%)	57 (61.3 %)
Flame, *n*(%)	30 (32.2 %)
Other, *n*(%)	6 (6.5 %)
Age	
Age median	2 years and 3 months
	(range 2 months–66 years)
Age 25th percentile	1 year and 4 months
Age 75th percentile	6 years and 8 months
≤4 years of age	*n* = 61 (65.6 %)
≥18 years of age	*n* = 9 (9.7 %)
TBSA burn	
TBSA median	2.0 %
	(range 0.5–36 % TBSA)
TBSA 25th percentile	1.00 %
TBSA 75th percentile	4.00 %
TBSA ≤5 %	*n* = 81 (87.1 %)
TBSA >10 %	*n* = 2 (2.2 %)
TBSA median adults	3.0 % (range 1–10 %)

Complications (Table 2) occurred in three patients: two patients (2.2 %) developed a folliculitis, scab formation was found in one patient (1.1 %). No chronic infection or alopecia developed in our patient population. No patient showed scar hypertrophy at the donor site.

**Table 2:** Outcome of the donor sites

Folliculitis, *n*(%)	2 (2.2)
Scab formation, *n*(%)	1 (1.1)
Alopecia, *n*(%)	0 (0)
Scar hypertrophy, *n*(%)	0 (0)
Healing of donor site	100 % < 14 days

All donor sites healed within 14 days.

## Discussion

This review shows a low complication rate of 3.2 % in 105 skin grafts from the scalp in 93 patients over a period of 7 years, without any lasting effects of these complications. This complication rate is comparable to rates found in literature [3–7, 11, 12], with fewer lasting complications. However, the majority of our population had a small TBSA burned, possibly decreasing the complication rate due to a smaller wound surface area with possibly smaller risk of infection. Kidd et al. [13] found a higher rate of hypertrophic scarring after burns in patients with a larger TBSA involved. Whether this is also true for complications after harvesting of the scalp is unclear: donor sites are surgical wounds, and thus sterile, while burns are traumatic wounds, and thus more likely to be contaminated.

Another explanation for the lack of alopecia and chronic folliculitis might be the thickness of our grafts. Our grafts are very thin—leaving the bulge stem cell region intact [9]—although a difference in thickness with earlier literature cannot be quantified, as mentioned above.

The graft thickness according to the readings on the dermatome is only an indication of the thickness of the graft. The graft thickness can also be influenced by the amount of pressure and the angle applied during harvesting. For a more accurate statement on the graft thickness, the measurements of the graft are needed. No standardised measuring device is available on market today. In our Burn Centre, we are currently developing such a device, to more accurately answer the question of the thickness of a graft compared to the setting of the dermatome and complications in wound healing of the donor site.

More precise data on the healing time of the donor site could not be obtained from the files due to the fact that most children were at home at the time of healing. Either the child was treated in the outpatient clinic, or, in case of clinical admission, had left the hospital at the fifth postoperative day when take of the transplant was judged to be good. Thus, the patient was at home when the donor site had healed and the alginate crust detached. When they were next evaluated at the outpatient clinic, a few days later by a medical professional, the exact date was not noted in the file, as it was only possible to objectively appraise whether the site had healed, not when.

Due to the application of the alginate dressing, postoperative care is virtually non-existent, thus interfering minimally with rehabilitation. We found that the donor site heals within 14 days, at which point it can be re-harvested if necessary [3, 5–7, 9, 11, 12]. In young children in particular, the scalp offers a large donor site surface as the head is relatively big. In previous literature, as well as in our own review, we find a low complication rate, with no lasting adverse effects in our hospital.

## Conclusions

Our study showed that the scalp is a reliable donor site, meeting most of the criteria proposed by Chang et al. Therefore, we propose the use of the scalp as a primary donor site in young children and in adults with facial burns, provided that the skin was harvested with an accurate dermatome by an experienced surgeon and that complications like folliculitis are treated immediately with the appropriate antiseptic.

### Ethics approval

No ethics approval was sought, because the study was performed retrospectively, with existing data, without any research intervention to/of the patient.

In The Netherlands, if patients are not subjected to study interventions (as is the case in a retrospective study), no medical-ethical approval is necessary according to the WMO (Wet Medisch-wetenschappelijk Onderzoek = Law of Medical/Scientific Research):

“Retrospective research/research with patient files does not fall under the scope of the WMO as the research subject is not physically involved in the research.” as quoted from the website of the Central Committee of Research involving human subjects. (http://www.ccmo.nl/en/file-research).

### Consent for publication

N/A.

## Abbreviations

SSG: split skin grafting
TBSA: total body surface area

## References

[1] Crawford BS (1964). An unusual donor site. Br J Plast Surg.

[2] Bach C-A (2012). The scalp or how to reduce the scarring associated with the harvesting of a split-thickness skin graft in head and neck surgery. Eur Ann Otorhinolaryngol Head Neck Dis.

[3] Mimoun M, Chaouat M, Picovski D, Serroussi D, Smarrito S (2006). The scalp is an advantageous donor site for thin-skin grafts: a report on 945 harvested samples. Plast Reconstr Surg.

[4] Weyandt GH, Bauer B, Berens N, Hamm H, Broecker E-B (2009). Split-skin grafting from the scalp: the hidden advantage. Dermatol Surg.

[5] Gyger D, Genin B, Bugmann P, Lironi A, Le Coultre C (1996). Skin harvesting on the scalp in children: utopia or reality. Eur J Pediatr Surg.

[6] Wyrzykowski D, Chrzanowska B, Czauderna P (2015). Ten years later - scalp still a primary donor site in children. Burns.

[7] Farina JA, Freitas FAS, Ungarelli LF, Rodrigues JM, Rossi LA (2010). Absence of pathological scarring in the donor site of the scalp in burns: an analysis of 295 cases. Burns.

[8] Martinot V, Mitchell V, Fevrier P, Duhamel A, Pellerin P (1994). Comparative study of split thickness skin grafts taken from the scalp and thigh in children. Burns.

[9] Jimenez F, Izeta A, Poblet E (2011). Morphometric analysis of the human scalp hair follicle: practical implications for the hair transplant surgeon and hair regeneration studies. Dermatol Surg.

[10] Philp L, Umraw N, Cartotto R (2012). Late outcomes after grafting of the severely burned face: a quality improvement initiative. J Burn Care Res.

[11] Barret JP, Dziewulski P, Wolf SE, Desai MH, Herndon DN (1999). Outcome of scalp donor sites in 450 consecutive pediatric burn patients. Plast Reconstr Surg.

[12] Chang L-Y, Yang J-Y, Chuang S-S, Hsiao C-W (1998). Use of the scalp as a donor site for large burn wound coverage: review of 150 patients. World J Surg.

[13] Kidd LR, Nguyen DQ, Lyons SC, Dickson WA (2013). Following up the follow up—long-term complications in paediatric burns. Burns.

